# Profile of fluency in spontaneous speech, reading, and retelling of texts by adults who stutter

**DOI:** 10.1590/2317-1782/20232022009en

**Published:** 2023-09-25

**Authors:** Samuel Lopes da Silva, Luciana Mendonça Alves, Denise Brandão de Oliveira e Britto

**Affiliations:** 1 Departamento de Fonoaudiologia, Faculdade de Medicina, Universidade Federal de Minas Gerais - UFMG - Belo Horizonte (MG), Brasil.

**Keywords:** Language and Hearing Sciences, Stuttering, Reading, Speech, Adult, Childhood-Onset Fluency Disorder

## Abstract

**Purpose:**

to describe the profile of fluency concerning the typology of disfluencies, speed, and frequency of disruptions in spontaneous speech, reading, and retelling; to compare the fluency profile in adults who stutter in spontaneous speech, reading, and retelling of text.

**Methods:**

The present work is a cross-sectional comparative study with a sample composed of 15 adults who stutter of both sexes, with higher education or equivalent to complete elementary school II. Samples were collected in the tasks of spontaneous speech, reading, and text retelling through video calls made individually with the participants. The first 200 syllables expressed in each task were transcribed and analyzed according to the Fluency Profile Assessment Protocol (FPAP). The study compared the frequency of common and stuttering disfluencies and the speed in the different tasks surveyed. The Kruskal & Wallis test was used together with Duncan's multiple comparisons test to compare the medians and verify possible differences between the tasks researched with a significance level of 5%.

**Results:**

The reading task presented a lower number of common disfluencies and a percentage of speech discontinuity about spontaneous speech and retelling tasks. No statistically significant differences were found between stuttering disfluencies in the three tasks surveyed.

**Conclusion:**

This study showed that there are differences in the occurrence of common disfluencies - hesitations, interjections, and revisions - and in the percentage of speech discontinuity during an oral reading of adults who stutter concerning spontaneous speech and text retelling.

## INTRODUCTION

Stuttering is a complex impairment of fluency, characterized by the presence of interruptions of speech that interfere with the smooth, continuous verbal flow of those who stutter. It has a multifactorial etiology with greater prevalence in men, and a relation to heredity in its emergence and development. There is consensus in the literature regarding the genetic factor in terms of increased risk of stuttering, as well as other still little understood factors^(1)^.

Impaired fluency has motor, neurological, emotional, and linguistic dimensions that affect an individual’s speed and flow of speech^(2)^. Breaks such as interruptions, repetitions, pauses, and prolongations among other types of disfluency can be observed in different cases of stuttering^(3)^.

Assessing fluency can be undertaken using clinical observation of the individual’s speech, applying protocols and instruments that qualitatively and quantitatively describe fluency, the events that impair the fluency of spontaneous speech, and other tasks where the individual uses their mouth^(3)^.

Reading, for instance, is an activity depending on a series of complex neurological and cognitive processes, in which fluency plays an important role. Efficient reading is directly related to the individual’s reading and speech fluency, with speed and precision of the number of words read per minute being important for academic, social, linguistic, and cognitive development among other competencies and abilities^(4,5)^. Effective understanding of written linguistic codes relies on having adequate production of reading in a smooth and regular way^(3,5)^.

Reading fluency is the ability to evenly, spontaneously, easily, and continuously read texts. It is characterized by an absence of failures in the automatic identification of words, adequate speed, rhythm, and prosody. It is crucial for effective reading and contributes to processes of understanding and expression of the content of messages. Therefore, we expect that individuals who stutter may present difficulties when carrying out reading activities such as reading aloud. Studies that seek to compare performance during reading tasks, in both adults who stutter and those who do not, have shown a reduction in reading disfluencies in adults who stutter^(6,7)^.

Being a broad topic of significant interest in scientific and clinical contexts, more research is necessary to help professionals and researchers in their respective fields of activity. Studies that compare spontaneous speech, reading aloud, and text-retelling tasks can help to highlight the differences and similarities between occurrences of common and stuttering disfluencies, as well as changes in speed in adults who stutter. Thus, this study adopted the hypothesis that adults who stutter can present differences in the frequency and duration of these disfluencies as well as speed between spontaneous speech, reading aloud, and retelling tasks.

Therefore, this study seeks to describe the fluency profile for spontaneous speech, reading, and retelling in adults who stutter and compare the fluency profile about the type and frequency of disfluencies and speed of speech.

## METHODS

This study was approved by the Research Ethics Committee of the Federal University of Minas Gerais (UFMG) under the CAAE registration number 26669319.9.0000.5149, assessment number 4.458.559.

It is a comparative, cross-sectional study with a sample consisting of 15 adults who stutter, recruited from support groups, sites, and social networks , and clinics and institutions focused on treating individuals who stutter. Data collection was undertaken remotely using the platform Zoom® observing social distancing measures during the Covid-19 pandemic period.

The inclusion criteria were being older than or equal to 18 years old, presenting persistent stuttering, having a minimum education level of complete primary school, and being a native speaker of Brazilian Portuguese. The exclusion criteria were being diagnosed with a psychiatric disorder, disease, or neurological condition, or presenting auditory or visual alterations that made reading texts and understanding instructions impossible. All participants signed the Informed Consent Form (ICF) and agreed with the terms of the study.

During each interview, a protocol considering the clinical history and complaints, difficulties about stuttering, and family history of stuttering was adopted. The speech, reading, and retelling samples were collected with video and audio recordings during the interviews using the platform mentioned above with the following order and procedure: spontaneous speech using a script (personal presentation, daily routine, and speech elicitation using a drawing, when necessary); reading a text aloud for analysis of reading fluency^(8)^, and retelling the same text. A transcription of the first 200 syllables from each sample for the fluency analysis was made. The reading and retelling samples were dealt with in the same manner as the spontaneous speech samples.

The speech, reading, and retelling samples were analyzed according to the types of common and stuttering disfluencies, frequency of interruptions, and speed of speech according to the Fluency Profile Assessment Protocol (FPAP)^(3)^. The FPAP includes an analysis of the transcriptions observing the occurrence of common disfluencies, stuttering disfluencies, per minute word and syllable flow, percentage of speech discontinuities, and stuttering disfluencies. Data collection and analysis were undertaken by the researchers.

The data were stored in a data bank using the ExcelⓇ software, version 2016. Statistical analysis was carried out with Statistical Package for Social SciencesⓇ (SPSS) software, version 24. The Shapiro & Wilk Test was used to assess the data probability distribution. The Kruskal & Wallis test was used together with Duncan’s Multiple Range Test to compare the medians and determine any possible differences between spontaneous speech, reading aloud, and retelling samples in terms of the type of disfluency and percentage of common and stuttering disfluencies. The level of significance used for all analyses was 5% with significant p-values highlighted in bold.

## RESULTS

The sample consisted of five (5) female participants (N%=33.33) with an average age of 32 and SD=3.41 and ten (10) male participants (N%=66.67) with an average age of 27.1 and SD=9.16. The average age for all participants was 28.7 years with SD=8.0. All participants had the minimum education necessary to participate in the study, distributed as follows: one (1) (N%=6.67) participant with complete primary education, five (5) (N%=23.33) participants with incomplete/complete high school education, and nine (9) (N%=60) participants with incomplete/complete university education.

Data analysis related to the clinical history and complaints of participants showed that 6.67% (N=1) of the patients presented speech or language problems during childhood, being described as phonological impairment, exchange of sounds in speech, and delayed speech development. All participants self-described as being stutters and 60% (N=9) reported other family members who stuttered. Of the total sample 75% of participants with family members who stuttered (N=6) reported that this included either their father, mother, and/or 1^st^ aunts/uncles.

Regarding the fluency analysis, the highest averages observed in different types of common disfluencies were in spontaneous speech and retelling, except for “unfinished words” and “word repetition” where the highest averages were in reading aloud (Table 1).

**Table 1:** Comparison of types of common and stuttering disfluencies in reading, spontaneous speech, and retelling

		Task	Minimum	Maximum	Median	Mean	Standard deviation	P Value*
Common disfluencies	Hesitations	Reading	0.0	20.0	0.0a	1.60	5.12	**< 0.001**
		Spontaneous speech	0.0	30.0	**4.0b**	7.13	8.16	
		Retelling	0.0	49.0	**3.0b**	6.80	12.01	
	Interjections	Reading	0.0	1.0	0.0a	0.13	0.35	**< 0.001**
		Spontaneous speech	0.0	18.0	**7.0b**	7.27	4.57	
		Retelling	2.0	21.0	**6.0b**	7.80	5.06	
	Revisions	Reading	0.0	1.0	0.0a	0.07	0.26	**0.009**
		Spontaneous speech	0.0	4.0	**1.0b**	1.13	1.36	
		Retelling	0.0	2.0	**1.0b**	0.73	0.80	
	Unfinished words	Reading	0.0	1.0	0.0	0.40	0.51	0.071
		Spontaneous speech	0.0	4.0	0.0	0.33	1.05	
		Retelling	0.0	1.0	0.0	0.07	0.26	
	Word repetition	Reading	0.0	5.0	1.0	1.47	1.55	0.064
		Spontaneous speech	0.0	4.0	0.0	0.87	1.41	
		Retelling	0.0	3.0	0.0	0.40	0.83	
	Segment repetition	Reading	0.0	6.0	1.0	1.40	1.55	0.607
		Spontaneous speech	0.0	8.0	1.0	1.47	2.10	
		Retelling	0.0	11.0	1.0	1.60	3.02	
	Total	Reading	0.0	25.0	3.0a	5.20	6.64	**< 0.001**
		Spontaneous speech	4.0	46.0	**14.0b**	18.13	12.28	
		Retelling	6.0	53.0	**13.0b**	17.40	11.81	
Stuttering disfluencies	Repetition of syllables	Reading	0.0	3.0	1.0	0.93	0.96	0.312
		Spontaneous speech	0.0	2.0	0.0	0.47	0.64	
		Retelling	0.0	2.0	0.0	0.53	0.74	
	Repetition of sounds	Reading	0.0	3.0	0.0	0.40	0.91	0.240
		Spontaneous speech	0.0	3.0	0.0	0.80	1.15	
		Retelling	0.0	8.0	0.0	0.60	2.06	
	Prolongations	Reading	0.0	9.0	1.0	1.87	2.75	0.134
		Spontaneous speech	0.0	8.0	3.0	3.27	2.31	
		Retelling	0.0	9.0	2.0	2.60	2.47	
	Blocks (*Bloqueios*)	Reading	0.0	16.0	0.0	2.93	4.85	0.337
		Spontaneous speech	0.0	17.0	1.0	2.73	4.50	
		Retelling	0.0	11.0	0.0	2.00	3.80	
	Pauses	Reading	0.0	6.0	2.0	2.07	1.67	0.442
		Spontaneous speech	0.0	5.0	3.0	2.87	1.81	
		Retelling	0.0	5.0	2.0	2.27	1.33	
	Repetition of monosyllabic words	Reading	0.0	3.0	0.0	0.40	0.83	0.100
		Spontaneous speech	0.0	0.0	0.0	0.00	0.00	
		Retelling	0.0	1.0	0.0	0.13	0.35	
	Intrusion	Reading	0.0	9.0	0.0	1.60	2.64	0.086
		Retelling	0.0	5.0	3.0	2.60	1.50	
	Total	Reading	2.0	19.0	11.0	10.20	6.59	0.511
		Spontaneous Speech	5.0	24.0	11.0	12.47	5.55	
		Retelling	3.0	22.0	8.0	10.67	5.96	

Note: The common disfluency of phrase repetition and the category of spontaneous speech for stuttering disfluency of intrusion were not evaluated because they are a constant

(*) Kruskal-Wallis test; ab - different letters indicate average differences (Duncan’s multiple range test); significant if p<0.050

Regarding stuttering disfluencies, higher averages were also observed in spontaneous speech and text retelling, except for “blocks” (“*bloqueios*”) and “repetition of monosyllabic words”.

When comparing common disfluencies - hesitations, interjections, revisions, and total - statistically significant differences were observed, indicating better performance in reading aloud in comparison with spontaneous speech and retelling.

Speech speed by per minute word flow presented a higher average in the spontaneous speech and retelling samples while reading aloud presented a higher average per minute syllable flow. The frequency of interruptions (disfluencies) showed a higher average for spontaneous speech and retelling, as well as in percentage for speech discontinuity and stuttering disfluencies. No statistically significant results were observed in the analysis of stuttering disfluencies and speech speed. The results for frequency of disfluencies showed statistical significance for the percentage of speech discontinuities (Table 2).

**Table 2:** Comparison between the speech speed and frequency of disfluencies in reading, spontaneous speech, and retelling

		Task	Minimum	Maximum	Median	Mean	Standard deviation	P Value*
Speed	Per minute word flow	Reading	48.6	143.8	86.2	90.38	30.12	0.527
		Spontaneous Speech	64.4	147.7	93.5	98.51	24.86	
		Retelling	65.5	147.1	94.1	98.79	23.01	
	Per-minute syllable flow	Reading	92.6	274.0	172.4	176.50	57.41	0.937
		Spontaneous Speech	109.3	241.0	172.4	169.61	40.88	
		Retelling	110.5	235.3	153.8	168.22	39.59	
Frequency of disfluencies	% of discontinuity and speech	Reading	1.0	18.5	6.5a	7.50	5.14	**0.001**
		Spontaneous Speech	9.0	26.5	**13.5b**	15.30	5.28	
		Retelling	7.0	33.0	**12.0b**	14.17	6.70	
	% of stuttering disfluencies	Reading	1.0	9.5	4.0	4.77	3.22	0.349
		Spontaneous Speech	2.5	12.0	5.5	6.23	2.78	
		Retelling	1.5	11.0	4.0	5.33	2.98	
		Spontaneous Speech	0.8	1.8	1.2	1.25	0.31	
		Retelling	0.9	1.8	1.3	1.25	0.30	

Note: The common disfluency of phrase repetition and the spontaneous speech category for the stuttering disfluency of intrusion were not assessed because they are a constant

(*) Kruskal-Wallis test; ab - different letters indicate average differences (Duncan’s multiple range test); significant when p<0.050

## DISCUSSION

The final study sample presented a prevalence of men at a proportion of two men for every woman (2:1) partially corroborating the literature, which highlights a prevalence in the male sex during the adult phase, but of four to five men for every woman (4-5:1)^(9)^. Regarding hereditary, the literature reported that around two or more family members of individuals with persistent developmental stuttering also presented stuttering^(10)^. In a study analyzing the family prevalence of stuttering, the authors observed a statistically significant difference between participants with family members who had first-degree relatives in comparison with second and third-degree relatives^(9)^. The data from this study agree with the literature, given that nine participants in this study reported having other family members who stutter. Of these, six mentioned first-degree relatives.

In this study, the adults presented close averages for per-minute syllable and word speeds for the assessed tasks, which suggests that the speech and reading speeds are similar for adult individuals who stutter. A study that undertook a comparative analysis of adults with and without stuttering for spontaneous speech and reading tasks in terms of time spent, and per-minute word and syllable flow in 15 adults with stuttering, also found no significant difference between these parameters, agreeing with the findings from this sample^(7)^. This suggests that the time necessary to carry out the retelling of text is also not directly influenced by stuttering since there was no statistically significant relationship between these parameters. For the frequency of disfluencies, common disfluencies, that is, the types that occur in the speech of both individuals with and without stuttering, presented statistically significant values when comparing reading with spontaneous speech and retelling (p<0.001). These results agree with the literature, given that they show a greater quantity of common disfluencies in spontaneous speech than in reading aloud^(7,11)^.

In this study, no statistically significant differences were observed in speed for reading aloud, spontaneous speech, or retelling. In studies carried out with school-aged children with stuttering, statistically significant differences were observed in reading and speaking speeds^(4,12)^. This finding leads us to speculate that the development of reading ability affects performance for reading speed. This explains the statistical differences observed in studies with school-aged children, different from those observed in this study with adults. The literature reports that reading speed tends to evolve with educational development, but reaches a plateau during the final years of primary school^(5,7,13-15)^.

It is notable that in the group studied no participant reported problems for reading development during the literacy phase and at the time of data collection. Such difficulties could be obstacles when developing reading ability, and an impairment for their fluency and consequently for their academic development. As such, given the absence of complaints related to reading development, we do not expect to observe significant differences between parameters related to reading speed in the sample studied. The bibliography highlights that the higher the level of education the better the level of reading fluency, which reinforces that reading speed is determined over time by abilities related to the development of reading ability^(4,8,13,16)^.

Regarding the analysis of the percentage of stuttering disfluencies, no statistically significant relationship between the spontaneous speech, reading, and retelling samples was observed (p<0.349). However, the percentages for speech discontinuity presented statistically significant differences when comparing reading with spontaneous speech and retelling. These results corroborate other studies that found that reading is a less demanding task in terms of the mechanisms involved in the linguistic and motor processes for speech, in addition to the elaboration of discourse, leading to a reduction in the occurrence of disfluencies^(7,17,18)^.

Another study compared the performance of adults who stutter during spontaneous speech and reading aloud^(7)^. For these adults, the authors mentioned that the presence of a higher number of stuttering disfluencies, such as blocks (*bloqueios*) and prolongations (*prolongamentos*) during spontaneous speech is explicable by the possible relationship between stuttering and basal ganglia functioning. The inadequate functioning of these structures in motor control of speech, associated with the temporal processing of the message to be expressed could, result in a greater occurrence of stuttering disfluencies during spontaneous speech^(19,20)^. Another explanation for the low occurrence of stuttering disfluencies while reading aloud is that the cerebral processing for this task involves other areas such as the occipital lobe and areas related to visual processing^(21)^. This suggests that reading has a positive effect on fluency, given that it modifies the neurophysiological and neurolinguistic mechanisms that directly involve speech production.

A comparison of the disfluency types found a statistically significant difference in the presence of common disfluencies - hesitations, interjections, and revisions - when comparing the reading-aloud task with spontaneous speech and retelling. The reading task presented the lowest values of these disfluencies, which agrees with the findings from the literature^(7,22,23)^. Notably, the literature also highlights that hesitations, interjections, and revisions are related to difficulty in formulating and elaborating statements during discourse, and lexical, semantic, and syntactic recall^(24)^. It also indicates that the occurrence of these disfluencies in adults who stutter is observed in greater numbers during spontaneous speech^(7)^. These findings also suggest that common disfluencies occur more often during the retelling task in comparison with reading due to its closeness to the spontaneous speech task. That is, retelling, similar to spontaneous speech, favors the occurrence of common and stuttering disfluencies given that they are speech tasks where the individual elaborates the discourse being expressed^(25,26)^. Notably, the retelling is directly affected by reading comprehension, and as reading ability develops together with the educational development of individuals, better reading comprehension is expected with improved literacy^(5,27)^.

Regarding study limitations, the distinction of individuals in terms of the degree of severity of stuttering was not undertaken. As such, while we found no studies in the literature that compared the spontaneous speech and text retelling tasks, given the absence of a categorization of the sample in terms of the degree of severity of stuttering, the variability between the number of occurrences of the various types of disfluencies did not provide a clearer understanding of the data variability when assessing the fluency profile for the three tasks studied. To better establish standards of comparison for the data, further research should be undertaken with a larger number of subjects, mainly for adults who stutter, in which there is a scarcity of studies that more thoroughly investigate stuttering in other speech tasks beyond spontaneous speech.

This study presents a novel comparison of the spontaneous speech profile with other tasks involving oral production, including reading aloud, with text retelling not being reported in other studies, in the literature considering the speech of adults who stuttered, up until the time of this research. Given the scarcity of studies about speech fluency in adults who stutter beyond those that investigated spontaneous speech, the present research fills an important gap in the literature.

## CONCLUSION

When analyzing the fluency profile of adults who stutter, this study found no difference in speed for the performance of spontaneous speech, reading aloud or retelling - in terms of per minute syllable or word flow. Reading aloud was different from spontaneous speech and retelling for the percentage of speech discontinuity, mainly when comparing the disfluency types of hesitation, interjection, and revision. No significant differences between the other disfluencies were observed, with greater similarity in the comparisons between spontaneous speech and retelling.

## References

[1] Nogueira PR, Oliveira CMC, Giacheti CM, Moretti-Ferreira D (2015). Gagueira desenvolvimental persistente familial: disfluências e prevalência. Rev CEFAC.

[2] Arcuri CF, Osborn E, Schiefer AM, Chiari BM (2009). Taxa de elocução de fala segundo a gravidade da gagueira. Pró-Fono R Atual Cient.

[3] Andrade CRF (2017). Adolescentes e adultos com gagueira: fundamentos e aplicações clínicas..

[4] Fiorin M, Ugarte CV, Capellini SA, Oliveira CMC (2015). Fluência da leitura e da fala espontânea de escolares: estudo comparativo entre gagos e não gagos. Rev CEFAC.

[5] Andrade AJL, Celeste LC, Alves LM (2019). Caracterização da fluência de leitura em escolares do Ensino Fundamental II.. Audiol Commun Res.

[6] Fuchs LS, Fuchs D, Hosp MK, Jenkins JR (2001). Oral Reading fluency as an indicator of reading competence: a theoretical, empirical, and historical analysis. Sci Stud Read.

[7] Pinto JCB, Schiefer AM, Ávila CRB (2013). Disfluências e velocidade de fala em produção espontânea e em leitura oral em indivíduos gagos e não gagos. Audiol Commun Res.

[8] Gentilini LKS, Andrade MEP, Basso FP, Salles JF, Martins-Reis VO, Alves LM (2020). Desenvolvimento de instrumento para avaliação coletiva da fluência e compreensão de leitura textual em escolares do ensino fundamental II. CoDAS.

[9] Oliveira CMC, Santos MJP, Giacheti CM, Ferrari C (2015). Prevalência familial e razão sexual da gagueira nos familiares de crianças gagas.

[10] Drayna D, Kilshaw J, Kelly J (1999). The sex ratio in familial persistent stuttering. Am J Hum Genet.

[11] Carvalho S (2014). Automonitoramento da fala de adultos que gaguejam. Distúrbios Comun.

[12] Pinto JS, Picoloto LA, Capellini SA, Palharini TA, Oliveira CMC (2021). Fluência e compreensão da leitura em escolares com e sem gagueira. CoDAS.

[13] Alves LM, Santos LFD, Miranda ICC, Carvalho IM, Ribeiro GL, Freire LSC (2021). Evolução da velocidade de leitura no Ensino Fundamental I e II. CoDAS.

[14] Cunha VLO, Silvia C, Capellini SA (2012). Correlação entre habilidades básicas de leitura e compreensão de leitura. Estud Psicol.

[15] Bottino AG, Correa J (2013). A compreensão leitora de jovens e adultos tardiamente escolarizados. Psicol Reflex Crit.

[16] Kawano CE, Kida ASB, Carvalho CAF, Ávila CRB (2011). Parâmetros de fluência e tipos de erros na leitura de escolares com indicação de dificuldades para ler e escrever. Rev Soc Bras Fonoaudiol.

[17] Wagner RK (2011). Relations among oral reading fluency, silent reading fluency, and reading comprehension: a latent variable study of firstgrade readers. Sci Stud Read.

[18] Costa JB, Ritto AP, Juste FS, Andrade CR (2017). Comparação da performance de fala em indivíduos gagos e fluentes. CoDAS.

[19] Alm PA (2004). Stuttering and the basal ganglia circuits: a critical review of possible relations. J Commun Disord.

[20] Alm PA (2006). Um novo referencial para compreender a gagueira: o modelo pré-motor duplo. IFA Congress.

[21] Dehaene S (2013). A aprendizagem da leitura modifica as redes corticais da visão e da linguagem verbal. Let Hoje.

[22] Roberts PM, Meltzer A, Wilding J (2009). Disfluencies in non-stuttering adults across sample lengths and topics. J Commun Disord.

[23] Buzzeti PBMM, Fiorin M, Martinelli NL, Cardoso ACV, Oliveira CMC (2016). Comparação da leitura de escolares com gagueira em duas condições de escuta: habitual e atrasada. Rev CEFAC.

[24] Silva P, da Silva P, Marconato E, Picoloto L, Vilela L, Oliveira C (2019). Pausas e hesitações na fala de adultos com e sem gagueira. Distúrb Comun.

[25] Hlavac J (2011). Hesitation and monitoring phenomena in bilingual speech: a consequence of code-switching or a strategy to facilitate its incorporation?. J Pragmatics.

[26] Andrade CRF, Martins VO (2011). Influencia del sexo y el nivel educativo en la fluidez del habla en personas adultas. Rev Logop Fon Audiol.

[27] Martins MA, Capellini SA (2014). Fluência e compreensão da leitura em escolares do 3º ao 5º ano do ensino fundamental. Estud Psicol (Campinas).

