# Supplementary material for: Profile of fluency in spontaneous speech, reading, and retelling of texts by adults who stutter
Source: Codas. 2023 Sep 25;35(5):e20220009. doi: 10.1590/2317-1782/20232022009en (PMC10688293; doi:10.1590/2317-1782/20232022009en)
Supplement: Translation

# Translation: Perfil da fluência na fala espontânea, leitura e no reconto de textos de adultos que gaguejam

**DOI:** 10.1590/2317-1782/20232022009pt

**Authors:** Samuel Lopes da Silva, Luciana Mendonça Alves, Denise Brandão de Oliveira e Britto

**Affiliations:** 1 Departamento de Fonoaudiologia, Faculdade de Medicina, Universidade Federal de Minas Gerais - UFMG - Belo Horizonte (MG), Brasil.

**Keywords:** Fonoaudiologia, Gagueira, Leitura, Fala, Adulto, Transtorno da Fluência com Início na Infância

## Abstract

**Objetivo:**

descrever o perfil da fluência em relação à tipologia das disfluências, velocidade e frequência de rupturas na fala espontânea, na leitura e no reconto; comparar o perfil da fluência em adultos que gaguejam na fala espontânea, na leitura e no reconto de texto.

**Método:**

O trabalho é um estudo transversal comparativo com amostra composta por 15 adultos que gaguejam de ambos os sexos, com formação superior ou equivalente ao ensino fundamental II completo. Foram coletadas amostras nas tarefas de fala espontânea, leitura e reconto de texto por meio de video chamadas realizadas individualmente. As 200 primeiras sílabas expressas de cada tarefa foram transcritas e analisadas segundo o Protocolo de Avaliação do Perfil da Fluência (PAPF). O estudo comparou a frequência das disfluências comuns e gagas e a velocidade nas tarefas pesquisadas. Adotou-se o teste de Kruskal & Wallis em conjunto com o de comparações múltiplas de Duncan para comparar as medianas e verificar possíveis diferenças entre as tarefas pesquisadas com nível de significância de 5%.

**Resultados:**

A tarefa de leitura apresentou menor número de disfluências comuns e percentual de descontinuidade de fala em relação às tarefas de fala espontânea e reconto. Não foram encontradas diferenças estatisticamente significantes entre as disfluências gagas nas três tarefas pesquisadas.

**Conclusão:**

Este trabalho mostrou que existem diferenças na ocorrência das disfluências comuns - hesitações, interjeições e revisões - e no percentual de descontinuidade de fala durante a leitura oral de adultos que gaguejam em relação à fala espontânea e ao reconto de texto.

## INTRODUÇÃO

A gagueira é um complexo transtorno da fluência, caracterizado pela presença de rupturas na fala que interferem no fluxo contínuo e suave da fluência verbal do indivíduo que gagueja. Possui etiologia multifatorial com maior prevalência no sexo masculino tendo uma relação com a hereditariedade para o seu surgimento e desenvolvimento. Há um consenso na literatura sobre a influência do fator genético aumentar o risco do surgimento da gagueira, além de outros fatores ainda não muito bem compreendidos^(1)^.

Esse transtorno da fluência possui diferentes traços motores, neurológicos, emocionais, e linguísticos que comprometem a velocidade e o fluxo da fala de um indivíduo^(2)^. Desse modo, rupturas como bloqueios, repetições, pausas, prolongamentos dentre outras tipologias de disfluências podem estar presentes nos diferentes quadros da gagueira^(3)^.

A avaliação da fluência pode ser realizada mediante a observação clínica da fala do indivíduo por meio de protocolos e instrumentos que descrevam a fluência, de forma qualitativa e quantitativa, os eventos que venham a comprometer a fluência da fala espontânea, assim como em outras tarefas em que o indivíduo faz uso da oralidade^(3)^.

Por sua vez, a leitura é uma atividade resultante de uma série de complexos processos neurológicos e cognitivos, na qual a fluência exerce um importante papel. A eficiência da leitura está diretamente relacionada a fluidez da fala e leitura do indivíduo, velocidade e precisão do número de palavras lidas por minuto, sendo importante para o desenvolvimento escolar, social, linguístico, cognitivo, dentre outras competências e habilidades^(4,5)^. Para uma boa compreensão do código linguístico escrito é importante que haja a produção adequada da leitura de forma suave e ritmada^(3,5)^.

A fluência leitora é a habilidade de ler textos de maneira suave, espontânea, fácil e contínua. Caracteriza-se por ausência de comprometimento na identificação automática de palavras, adequada velocidade, ritmo e prosódia. É fundamental para a leitura eficiente e contribui para os processos de entendimento e expressão do conteúdo da mensagem. Logo é esperado que indivíduos que gaguejam possam apresentar dificuldades na execução de atividades leitoras como ler em voz alta. Estudos que se destinam a comparar o desempenho em tarefas de leitura, em adultos que gaguejam e adultos que não gaguejam, têm demonstrado uma diminuição das disfluências na leitura nos adultos com gagueira^(6,7)^.

Por ser um tema abrangente e de amplo interesse no meio clínico e científico, mais pesquisas são necessárias para auxiliar profissionais e pesquisadores em suas áreas de atuação. Estudos que comparam as tarefas de fala espontânea, leitura oral e o reconto de textos podem contribuir para investigar as diferenças ou semelhanças de ocorrência de disfluências comuns e gagas, além de mudanças na velocidade em adultos que gaguejam. Dessa forma, esse estudo parte da hipótese de que adultos que gaguejam possam apresentar diferenças quanto a frequência e duração das disfluências assim como na velocidade entre as tarefas de fala espontânea, leitura oral e reconto.

Assim, este estudo tem como objetivo descrever o perfil da fluência na fala espontânea, na leitura e no reconto de texto de adultos que gaguejam e comparar o perfil da fluência em relação à tipologia e frequência das disfluências e velocidade de fala.

## MÉTODO

Este estudo foi aprovado pelo Comitê de Ética em Pesquisa da Universidade Federal de Minas Gerais (UFMG) sob o número de registro CAAE 26669319.9.0000.5149, parecer número 4.458.559.

Trata-se de um estudo transversal comparativo com amostra composta por 15 adultos que gaguejam convidados em grupos de apoio à gagueira, *sites*, redes sociais, ambulatórios e instituições voltadas para o atendimento de indivíduos que gaguejam. A coleta dos dados foi realizada de forma remota pela plataforma Zoom® respeitando as medidas de distanciamento social durante o período da pandemia provocada pelo vírus Covid-19.

Os critérios de inclusão foram: possuir idade maior ou igual a 18 anos, apresentar gagueira persistente, escolaridade mínima do ensino fundamental completo e ser falante nativo do português brasileiro. Quanto aos critérios de exclusão foram estabelecidos: presença de diagnóstico de desordens psiquiátricas, doença ou transtorno neurológico, apresentar alterações visuais e auditivas que impossibilitassem a leitura de textos e compreensão de comandos. Todos os participantes assinaram Termo de Consentimento Livre e Esclarecido (TCLE) e concordaram com os termos da pesquisa.

Durante cada entrevista foi utilizado com os participantes um protocolo de história clínica para coleta de dados referentes à queixa, dificuldades com relação à gagueira e antecedentes familiares para a gagueira. As amostras de fala, leitura e reconto foram coletadas com registro em áudio e vídeo durante a entrevista por meio da própria plataforma mencionada com a seguinte ordem e procedimento: fala espontânea: uso de roteiro elaborado (apresentação pessoal, rotina diária e eliciação da fala por meio de figura, quando necessário); leitura de texto para análise da fluência leitora^(8)^ e reconto do próprio texto. Foi realizada a transcrição literal das primeiras 200 sílabas de cada amostra para análise da fluência. Ressalta-se que as amostras de leitura e reconto foram tratadas da mesma forma que as amostras de fala espontânea.

As amostras de fala, de leitura e do reconto foram analisadas segundo a tipologia das disfluências gagas e comuns, frequência de rupturas e velocidade de fala de acordo com o Protocolo de Avaliação do Perfil da Fluência (PAPF)^(3)^. O PAPF consiste na análise das transcrições observando a ocorrência das disfluências comuns, disfluências gagas, fluxo de palavras por minuto e fluxo de sílabas por minuto, porcentagens de descontinuidade de fala e de disfluências gagas. A coleta e análise das amostras foram realizadas pelos próprios pesquisadores.

Os dados foram armazenados em um banco de dados por meio do *software* ExcelⓇ, versão 2016. A análise estatística foi realizada com o *software* Statistical Package for Social SciencesⓇ (SPSS), versão 24. Para avaliar a distribuição de probabilidade dos dados foi utilizado o teste de Shapiro & Wilk. Adotou-se o teste de Kruskal & Wallis em conjunto com o de comparações múltiplas de Duncan para comparar as medianas e verificar possíveis diferenças entre as amostras de fala espontânea, leitura e reconto quanto a tipologias das disfluências e porcentagem de disfluências comuns e gagas. O nível alfa de significância utilizado em todas as análises foi de 5% com valores de p significantes destacados em negrito.

## RESULTADOS

A amostra foi composta por cinco (5) participantes do sexo feminino (N%= 33,33) com média de idade de 32 anos e DP= 3,41 e dez (10) do sexo masculino (N%= 66,67) com média de idade de 27,1 anos e DP= 9,16. A média de idade entre todos os participantes foi de 28,7 anos com DP= 8,0. Todos os participantes possuíam o grau de escolaridade mínimo para participar do estudo distribuídos da seguinte forma: um (1) participante (N%= 6,67) com ensino fundamental completo, cinco (5) (N%= 23,33) ensino médio incompleto/completo e nove (9) (N%= 60) apresentaram ensino superior incompleto/completo.

A análise dos dados referentes à história clínica e a queixa dos participantes mostrou que 6,67% (N=1) dos pacientes apresentou problema de fala e ou linguagem durante a infância, sendo descrito como transtorno fonológico, troca de sons na fala, e atraso do desenvolvimento de fala. Todos os participantes se intitularam pessoas que gaguejam e 60% (N=9) relataram gagueira entre outros membros da família. Do total da amostra 75% dos participantes que possuíam familiares com gagueira (N=6) relataram que pai, mãe e/ou tios de 1º grau determinam o parentesco.

Em relação às análises da fluência observa-se que as maiores médias encontradas nos diferentes tipos de disfluências comuns foram na fala espontânea e no reconto, com exceção das “palavras não terminadas” e “repetição de palavras”, cuja maior média foi na leitura (Tabela 1).

**Tabela 1:** Comparação entre as tipologias das disfluências comuns e gagas na leitura, fala espontânea e reconto

		Tarefa	Mínimo	Máximo	Mediana	Média	Desvio padrão	Valor p*
Disfluências comuns	Hesitações	Leitura	0,0	20,0	0,0a	1,60	5,12	**< 0,001**
		Fala espontânea	0,0	30,0	**4,0b**	7,13	8,16	
		Reconto	0,0	49,0	**3,0b**	6,80	12,01	
	Interjeições	Leitura	0,0	1,0	0,0a	0,13	0,35	**< 0,001**
		Fala espontânea	0,0	18,0	**7,0b**	7,27	4,57	
		Reconto	2,0	21,0	**6,0b**	7,80	5,06	
	Revisões	Leitura	0,0	1,0	0,0a	0,07	0,26	**0,009**
		Fala espontânea	0,0	4,0	**1,0b**	1,13	1,36	
		Reconto	0,0	2,0	**1,0b**	0,73	0,80	
	Palavras não terminadas	Leitura	0,0	1,0	0,0	0,40	0,51	0,071
		Fala espontânea	0,0	4,0	0,0	0,33	1,05	
		Reconto	0,0	1,0	0,0	0,07	0,26	
	Repetição de palavras	Leitura	0,0	5,0	1,0	1,47	1,55	0,064
		Fala espontânea	0,0	4,0	0,0	0,87	1,41	
		Reconto	0,0	3,0	0,0	0,40	0,83	
	Repetição de segmentos	Leitura	0,0	6,0	1,0	1,40	1,55	0,607
		Fala espontânea	0,0	8,0	1,0	1,47	2,10	
		Reconto	0,0	11,0	1,0	1,60	3,02	
	Total	Leitura	0,0	25,0	3,0a	5,20	6,64	**< 0,001**
		Fala espontânea	4,0	46,0	**14,0b**	18,13	12,28	
		Reconto	6,0	53,0	**13,0b**	17,40	11,81	
Disfluências gagas	Repetição de sílabas	Leitura	0,0	3,0	1,0	0,93	0,96	0,312
		Fala espontânea	0,0	2,0	0,0	0,47	0,64	
		Reconto	0,0	2,0	0,0	0,53	0,74	
	Repetição de sons	Leitura	0,0	3,0	0,0	0,40	0,91	0,240
		Fala espontânea	0,0	3,0	0,0	0,80	1,15	
		Reconto	0,0	8,0	0,0	0,60	2,06	
	Prolongamentos	Leitura	0,0	9,0	1,0	1,87	2,75	0,134
		Fala espontânea	0,0	8,0	3,0	3,27	2,31	
		Reconto	0,0	9,0	2,0	2,60	2,47	
	Bloqueios	Leitura	0,0	16,0	0,0	2,93	4,85	0,337
		Fala espontânea	0,0	17,0	1,0	2,73	4,50	
		Reconto	0,0	11,0	0,0	2,00	3,80	
	Pausas	Leitura	0,0	6,0	2,0	2,07	1,67	0,442
		Fala espontânea	0,0	5,0	3,0	2,87	1,81	
		Reconto	0,0	5,0	2,0	2,27	1,33	
	Repetição de palavra monossilábica	Leitura	0,0	3,0	0,0	0,40	0,83	0,100
		Fala espontânea	0,0	0,0	0,0	0,00	0,00	
		Reconto	0,0	1,0	0,0	0,13	0,35	
	Intrusão	Leitura	0,0	9,0	0,0	1,60	2,64	0,086
		Reconto	0,0	5,0	3,0	2,60	1,50	
	Total	Leitura	2,0	19,0	11,0	10,20	6,59	0,511
		Fala espontânea	5,0	24,0	11,0	12,47	5,55	
		Reconto	3,0	22,0	8,0	10,67	5,96	

Nota: A disfluência comum de repetição de frases e a categoria de fala espontânea da disfluência gaga de intrusão não foram avaliadas porque são uma constante

(*) Teste de Kruskal-Wallis; ab - letras diferentes indicam diferenças medianas (teste de comparações múltiplas de Duncan); significativo se p<0,050

Quanto às disfluências gagas também se observou maiores médias na fala espontânea e no reconto do texto, exceto para os “bloqueios” e a "repetição de palavras monossilábicas”.

Ao se comparar as disfluências comuns - hesitações, interjeições, revisões e total - observa-se que os resultados foram estatisticamente significantes, caracterizando melhor desempenho na leitura em relação à fala espontânea e ao reconto.

A velocidade de fala apresentou maior média na amostra de fala espontânea e no reconto para o fluxo de palavras por minuto, porém a leitura apresentou maior média para o fluxo de sílabas por minuto. A frequência de rupturas (disfluências) obteve maior média de fala espontânea e reconto, assim como nos percentuais de descontinuidade de fala e de disfluências gagas. Em relação à análise das disfluências gagas e da velocidade de fala não foram encontrados resultados estatisticamente significantes. Quanto à frequência das disfluências os resultados apontam significância estatística no percentual de descontinuidade de fala (Tabela 2).

**Tabela 2:** Comparação entre a velocidade de fala e a frequência das disfluências na leitura, fala espontânea e reconto

		Tarefa	Mínimo	Máximo	Mediana	Média	Desvio padrão	Valor p*
Velocidade	fluxo de palavras por minuto	Leitura	48,6	143,8	86,2	90,38	30,12	0,527
		Fala espontânea	64,4	147,7	93,5	98,51	24,86	
		Reconto	65,5	147,1	94,1	98,79	23,01	
	fluxo de sílabas por minuto	Leitura	92,6	274,0	172,4	176,50	57,41	0,937
		Fala espontânea	109,3	241,0	172,4	169,61	40,88	
		Reconto	110,5	235,3	153,8	168,22	39,59	
Frequência das disfluências	% de descontinuidade de fala	Leitura	1,0	18,5	6,5a	7,50	5,14	**0,001**
		Fala espontânea	9,0	26,5	**13,5b**	15,30	5,28	
		Reconto	7,0	33,0	**12,0b**	14,17	6,70	
	% de disfluências gagas	Leitura	1,0	9,5	4,0	4,77	3,22	0,349
		Fala espontânea	2,5	12,0	5,5	6,23	2,78	
		Reconto	1,5	11,0	4,0	5,33	2,98	
		Fala espontânea	0,8	1,8	1,2	1,25	0,31	
		Reconto	0,9	1,8	1,3	1,25	0,30	

Nota: A disfluência comum de repetição de frases e a categoria de fala espontânea da disfluência gaga de intrusão não foram avaliadas porque são uma constante

(*) Teste de Kruskal-Wallis; ab - letras diferentes indicam diferenças medianas (teste de comparações múltiplas de Duncan); significativo se p<0,050

## DISCUSSÃO

A amostra final deste estudo apresentou prevalência do sexo masculino na proporção de dois homens para cada mulher (2:1) corroborando parcialmente a literatura que aponta que na fase adulta a prevalência é do sexo masculino, mas de quatro a cinco homens para cada mulher (4-5:1)^(9)^. Com relação ao aspecto hereditário, a literatura relata que em torno de dois ou mais familiares de pessoas com gagueira desenvolvimental persistente também apresentam gagueira^(10)^. Em um estudo que analisou a prevalência familiar da gagueira os autores mostraram que existe uma diferença estatisticamente significante entre os familiares que possuem parentesco de primeiro grau em relação aos de segundo e terceiro grau^(9)^. Os dados deste trabalho corroboram a literatura, uma vez que nove participantes deste estudo relataram apresentar outros membros na família que gaguejam e destes, seis participantes mencionaram parentes de primeiro grau.

Neste estudo, os adultos apresentaram médias quanto à velocidade de sílabas e palavras por minuto próximas entre as tarefas pesquisadas, o que sugere que a velocidade de fala e leitura são similares na pessoa adulta que gagueja. Em um estudo que realizou uma análise comparativa entre adultos com e sem gagueira nas tarefas de fala espontânea e leitura quanto ao tempo gasto, fluxo de palavras e sílabas por minuto de 15 adultos com gagueira também mostrou que não há diferença significativa entre esses parâmetros corroborando os achados dessa amostra^(7)^. Esse achado sugere que o tempo necessário para a realização do reconto do texto também não é influenciado diretamente pela gagueira pois não houve uma relação estatisticamente significante nesses parâmetros. Quanto à frequência das disfluências observa-se que as disfluências comuns, ou seja, as tipologias que ocorrem na fala de indivíduos com e sem gagueira, apresentam valores estatisticamente significantes ao se comparar a leitura com a fala espontânea e o reconto (p<0,001). Esses resultados corroboram a literatura, uma vez que mostram que a quantidade de disfluências comuns é maior na fala espontânea em relação à leitura^(7,11)^.

Neste estudo não foram encontradas diferenças estatisticamente significantes na velocidade da leitura, da fala espontânea e do reconto. Em estudos realizados com escolares com gagueira, diferenças estatisticamente significantes foram observadas na velocidade de fala e de leitura^(4,12)^. Tal achado leva a especular que o desenvolvimento da habilidade leitora influencia no desempenho da velocidade de leitura. Este resultado justifica tais diferenças estatísticas encontradas nos estudos com escolares, diferentemente do observado nesta pesquisa com adultos. A literatura relata que a velocidade de leitura tende a evoluir com o avanço da escolaridade, mas atinge um patamar de estabilidade dos anos finais do ensino fundamental II^(5,7,13-15)^.

Destaca-se o fato de que no grupo estudado nenhum participante relatou apresentar problemas quanto ao desenvolvimento da leitura durante a fase de alfabetização e no momento da coleta dos dados. Tais dificuldades podem representar obstáculos no desenvolvimento na habilidade de leitura, com comprometimento da sua fluência e consequentemente no percurso acadêmico. Logo, na ausência de queixas relacionadas ao desenvolvimento da leitura, não é esperado observar diferenças significativas entre os parâmetros relacionados à velocidade de leitura na amostra estudada. A bibliografia aponta que quanto maior o nível de escolaridade melhor será a habilidade fluência leitora, o que reforça o fato de que a velocidade de leitura é determinada pelas habilidades relacionadas ao desenvolvimento da habilidade leitora ao longo do tempo^(4,8,13,16)^.

Quanto à análise do percentual das disfluências gagas não houve relação estatisticamente significante entre as amostras de fala espontânea, leitura oral e reconto (p<0,349). Entretanto, os percentuais de descontinuidade de fala apresentaram diferenças estatisticamente significantes quando se comparou a leitura com a fala espontânea e o reconto. Esses resultados corroboram outros estudos que mostram que a leitura é uma tarefa com menor exigência quanto aos mecanismos envolvidos no processo linguístico e motor da fala além da elaboração do discurso, ocasionando a diminuição da ocorrência de disfluências^(7,17,18)^.

Outro estudo comparou a performance de adultos que gaguejam durante a fala espontânea e a leitura oral^(7)^. Nele os autores mencionaram que a presença de um maior número das disfluências gagas, como os bloqueios e os prolongamentos, durante a fala espontânea é justificada pela possível relação entre a gagueira e o funcionamento dos núcleos da base. O funcionamento inadequado dessas estruturas para o controle motor da fala, associado ao processamento temporal da mensagem a ser expressa resultaria em uma maior ocorrência das disfluências durante a fala espontânea^(19,20)^. Outra justificativa para a baixa ocorrência de disfluências gagas durante a leitura oral é que o processamento cerebral desta tarefa envolve outras áreas como a occipito temporal e áreas relacionadas ao processamento visual^(21)^. Dessa forma, sugere-se que a leitura provoca um efeito positivo na fluência, uma vez que ela modifica os mecanismos neurofisiológicos e neurolinguísticos que envolvem diretamente a produção da fala.

A comparação entre as tipologias das disfluências mostra que há uma diferença estatisticamente significante quanto a presença das disfluências comuns - hesitações, interjeições e revisões - entre as tarefas de leitura oral quando comparada com a fala espontânea e reconto. A tarefa de leitura foi a que apresentou menores valores dessas disfluências, o que vai ao encontro dos achados da literatura^(7,22,23)^. Destaca-se que a literatura também aponta que as hesitações, interjeições e revisões estão relacionadas com a dificuldade na formulação e elaboração de enunciados durante o discurso, resgate lexical, sintático e semântico^(24)^. Assim como refere também que a ocorrência dessas disfluências em adultos que gaguejam são observadas em maior número na fala espontânea^(7)^. Esses achados sugerem ainda que as disfluências comuns ocorreram em maior número na tarefa do reconto em relação à leitura pelo fato da sua aproximação com a tarefa da fala espontânea. Ou seja, o reconto, assim como a fala espontânea, favorece a ocorrência das disfluências comuns e gagas levando em consideração que são tarefas de fala em que o indivíduo precisa elaborar o discurso a ser expresso^(25,26)^. Destaca-se que o reconto é influenciado diretamente pela compreensão leitora, e de acordo com o desenvolvimento da habilidade de leitura em relação ao avanço da escolaridade dos indivíduos, espera-se uma melhor compreensão leitora com o avanço da alfabetização^(5,27)^.

Como limitações deste estudo ressalta-se que não foi realizada distinção dos indivíduos quanto ao grau de gravidade da gagueira e na literatura não foram encontrados estudos que comparam a tarefa de fala espontânea e o reconto de textos. Contudo, como não foi realizada uma categorização da amostra quanto ao nível de gravidade da gagueira, a variabilidade entre o número de ocorrências das tipologias das disfluências não possibilitou um melhor entendimento quanto a variabilidade dos dados no que se refere a avaliação do perfil da fluência nas três tarefas pesquisadas. Para estabelecer melhores padrões de comparação desses dados, outras pesquisas devem ser realizadas com maior número de sujeitos, principalmente com adultos que gaguejam, onde há uma escassez de estudos que investiguem mais a fundo a gagueira em outras tarefas de fala que não somente a fala espontânea.

Como avanço, o estudo comparou o perfil da fala espontânea em outras tarefas que envolvem a produção oral, como a leitura oral, sendo que o reconto de textos não foi descrito na literatura na fala de adultos que gaguejam até o momento em que essa pesquisa foi realizada. Mediante a escassez de estudos quanto à fluência na fala de adultos que gaguejam além da fala espontânea, o presente trabalho preenche uma importante lacuna na literatura.

## CONCLUSÃO

Os resultados evidenciaram que na análise do perfil da fluência de adultos que gaguejam não há diferença no desempenho durante a fala espontânea, leitura oral e reconto quanto à velocidade - fluxo de sílabas e palavras por minuto. A leitura oral se diferenciou da fala espontânea e do reconto quanto à porcentagem da descontinuidade de fala, principalmente quando comparadas as tipologias das disfluências de hesitações, interjeições e revisões. Não foram encontradas diferenças significativas entre as demais disfluências, com maior semelhança nas comparações entre a fala espontânea e o reconto.
